# Expanding the Potential of Circular RNA (CircRNA) Vaccines: A Promising Therapeutic Approach

**DOI:** 10.3390/ijms26010379

**Published:** 2025-01-04

**Authors:** Tian Bu, Ziyu Yang, Jian Zhao, Yanmei Gao, Faxiang Li, Rong Yang

**Affiliations:** 1 State Key Laboratory of Developmental Biology of Freshwater Fish, Engineering Research Center of Polyploid Fish Reproduction and Breeding of the State Education Ministry, College of Life Sciences, Hunan Normal University, Changsha 410081, China; butian@hunnu.edu.cn (T.B.); 202220142856@hunnu.edu.cn (Z.Y.); z3081386795@hunnu.edu.cn (J.Z.); 202320142793@hunnu.edu.cn (Y.G.); 2 MOE Key Laboratory of Rare Pediatric Diseases, Center for Medical Genetics, School of Life Sciences, Central South University, Changsha 410081, China

**Keywords:** circular RNAs, circRNA vaccine, infectious diseases, tumor

## Abstract

In recent years, circular RNAs (circRNAs) have garnered significant attention due to their unique structure and function, positioning them as promising candidates for next-generation vaccines. The circRNA vaccine, as an RNA vaccine, offers significant advantages in preventing infectious diseases by serving as a vector for protein expression through non-canonical translation. Notably, circRNA vaccines have demonstrated enduring antigenic expression and generate a larger percentage of neutralizing antibodies compared to mRNA vaccines administered at the same dosage. Furthermore, circRNA vaccines can elicit robust cellular and humoral immunity, indicating their potential for tumor vaccine development. However, certain challenges must be addressed to facilitate the widespread use of circRNA vaccines in both infectious disease prevention and tumor treatment. These challenges include the low efficiency of linear RNA circularization, the suboptimal targeting of delivery systems, and the assessment of potential side effects. This work aims to describe the characteristics and functions of circRNAs, elucidate the mechanism behind circRNA vaccines, and discuss their applications in the prevention of infectious diseases and the treatment of tumors, along with their potential future applications.

## 1. Introduction

### 1.1. Characteristics of circRNAs

**Figure 1:**
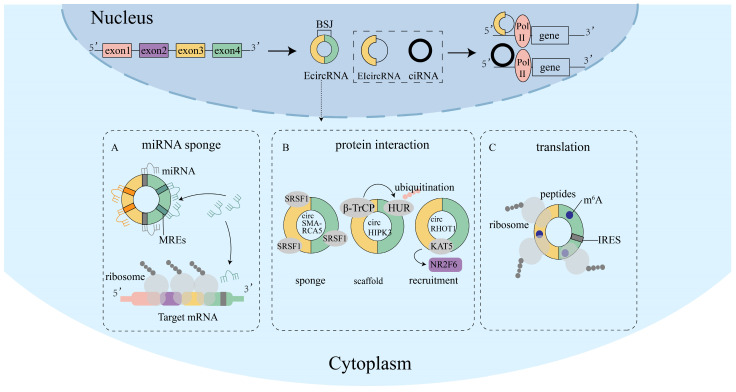
Functions of circRNAs. CircRNAs are a type of covalently closed circular single-stranded RNA generated by special variable splicing, and they primarily originate from exons. CircRNAs that arise from exons function in the cytoplasm, while those containing introns play a regulatory role in the nucleus. (**A**) CircRNAs with MRES can act as sponges for miRNAs in the cytoplasm, competing with target genes for binding to miRNAs and thereby inhibiting the expression of those genes. (**B**) CircRNAs interact with proteins in several ways. First, they can bind to RBPs, regulating the functions of these proteins. Second, circRNAs can serve as scaffolds for proteins, influencing their interactions. For example, circHIPK3 acts as a protein scaffold, promoting the binding of E3 ubiquitin ligase β-TrCP and HuR, leading to HuR’s ubiquitination and degradation. Third, circRNAs can recruit proteins to specific locations. For instance, circRHOT1 transports KAT5 into the nucleus to interact with the promoter of NR2F6. (**C**) circRNAs can serve as templates for protein translation when they possess m^6^A modifications or IRES. MREs, microRNA response elements; RBPs, RNA-binding proteins; IRES, internal ribosome entry site; m^6^A, N6-Methyladenosine; EcircRNA, exonic circRNAs; ciRNA, intronic circRNAs; EIcircRNA, exon-intronic circRNAs; BSJ, back-splicing junction.

CircRNAs are a type of non-coding RNA that lack a 5′-end cap and a 3′-end poly(A) tail. They are generated in a circular form through specific alternative splicing and can be categorized into exonic circRNAs (EcircRNA), intronic circRNAs (ciRNA), and exon-intronic circRNAs (EIcircRNA) (Figure 1) [1]. The circularization of linear RNA requires the involvement of RNA-binding proteins (RBPs) or Alu elements, which ensure the close proximity of the regions that need to be circularized, resulting in the formation of a head-to-tail circular RNA [2]. An analysis of metazoan transcriptome data has revealed that a significant number of transposable elements produce substantial quantities of circRNA [3,4]. Initially considered as by-products of erroneous splicing and underappreciated [5], the emergence of high-throughput sequencing has led to the discovery of a large number of physiologically functional circRNAs [6]. CircRNAs, which are highly evolutionarily conserved [7], are prevalent in eukaryotic cells and are predominantly located in the cytoplasm or exosomes, with only a small number present in the nucleus [8]. The circular structure of circRNAs provides them with greater stability compared to linear mRNAs, as they are resistant to degradation by RNase R and exhibit a half-life that is approximately 10 times longer than that of linear RNA upon treatment with actinomycin D. Furthermore, circRNAs exhibit distinct expression patterns across diverse cell types, cellular growth stages, and disease types.

### 1.2. Functions of circRNAs

The functions of circRNAs have been extensively explored, revealing their involvement not only in regulatory processes but also in protein encoding, thereby playing a significant role in the regulation of physiological and pathological mechanisms within organisms. In particular, circRNAs have been extensively studied as competing endogenous RNAs (ceRNAs) that function as miRNA sponges (Figure 1A). For instance, Hedgehog-Gli1-induced exosome circ-0011536 acts as a miR-451a sponge, promoting the expression of versatile growth factor (VGF) and activating Hedgehog signaling to regulate pancreatic ductal adenocarcinoma [9], and circANAPC7 acts as a sponge for miR-373, which inhibits tumor growth and muscle atrophy in vitro and in vivo [10]. Additionally, the interaction with proteins is an important regulatory function of circRNAs (Figure 1B). CircRNAs can interact with proteins by acting as protein sponges [11], facilitating protein–protein interactions as scaffolds [12], or recruiting proteins to modulate their localization [13]. For instance, circSMARCA5 can act as a sponge for serine/arginine-rich splicing factor 1 (SRSF1) protein, regulating vascular endothelial growth factor (VEGFA) and thus affecting glioblastoma multiforme [14]. Furthermore, a study indicated that circPVT1 functions as a scaffold for β-TrCP, an E3 ubiquitin ligase, influencing the degradation of c-Myc and, consequently, the invasion and migration of nasopharyngeal carcinoma (NPC) cells [15]. These interactions, in turn, affect gene transcription and regulate the organism’s physiological functions. While only a few circRNAs possess protein encoding capabilities (Figure 1C), their translation does not rely on the n7-methylguanosine (m^7^G) cap structure. Instead, circRNAs with an internal ribosomal entry site (IRES) or N6-Methyladenosine (m^6^A) modification can initiate translation through a rolling circle amplification mechanism [16]. IRES is a sequence located in the 5′ UTR of linear mRNA that directly recruits ribosomes [17], and m^6^A is the most abundant modification in eukaryotic cells, it binds eukaryotic initiation factor 3 (eIF3) to initiate translation. The coding function of circRNAs endows them with the potential to serve as protein drug molecules, and several circRNAs with coding capabilities have been found to play important roles in disease onset and progression. For instance, a research reported that the E7 oncoprotein encoded by circE7 from oncogenic human papillomavirus (HPV) could regulate the growth of cancer cells. Knocking down the expression of E7cicrRNA allowed them to inhibit the growth of cancer cells and slow down the growth of cancer tissue in tumor xenograft experiments [18]. The diverse functions of circRNAs position them as regulators in various biological pathways, including cellular homeostasis, viral infections, immune responses, aging, and death [19,20,21,22,23].

### 1.3. The Functional Mechanism of circRNA Vaccines

**Figure 2:**
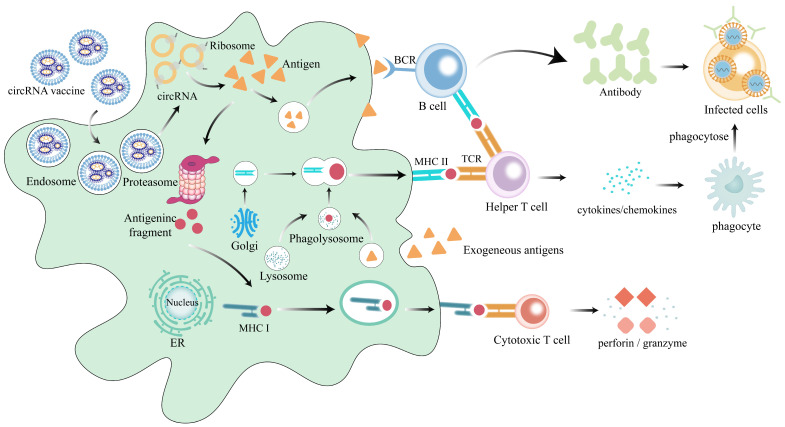
The antigens encoded by circular RNA vaccines activate immune responses. CircRNAs are encapsulated by LNPs and enter cells in endosomal form, where they are translated by ribosomes in the cytoplasm into antigenic proteins, which can be cleared of pathogens by both cellular and humoral immunity. Cellular immunity: Antigenic proteins in the cytoplasm are broken down into small antigenic fragments in the proteasome and are presented to cytotoxic T cells (CD8^+^ cells) via MHC I. These antigenic fragments stimulate CD8^+^ cells to secrete molecules, such as perforins and granzymes, which ultimately destroy the infected cells. Humoral immunity: The secreted antigen can be taken up by APCs and digested in the endosomes, then presented to CD4^+^ T cells via MHC II, which provide stimulatory signals such as CD40L to stimulate them to differentiate into plasma cells to produce antibodies, thus, exerting humoral immunity. Additionally, antigens can also directly stimulate B cells to initiate humoral immunity. LNPs, lipid nanoparticles; APCs, antigen-presenting cells; MHC I, major histocompatibility complex I; MHC II, major histocompatibility complex II; CD40L, cluster of differentiation 40 ligand.

CircRNA vaccines are administered through injection into tissues, and their mechanism of action is as follows (Figure 2). When delivered using lipid nanoparticle (LNP) systems, circRNA vaccines adhere to the cell membrane through electrostatic interactions and are subsequently taken up by the cell through endocytosis, forming endosomes [24]. In the acidic environment of the endosome, the LNPs undergo protonation, leading to fusion with the endosomal membrane [25], which results in the release of the circRNA encoding the antigen into the cytoplasm. The circRNAs are then translated into antigenic proteins by ribosomes. These proteins are processed into small antigenic peptides by proteasomes and presented to CD8^+^ T cells via major histocompatibility complex I (MHC I). Activated CD8^+^ T cells secrete cytokines, initiating the regulation of adaptive immunity and contributing to the elimination of infected cells [26]. Moreover, the full-length antigenic proteins encoded by circRNAs can be secreted into the extracellular space via the circulatory system, where they are directly recognized by B cell receptors on B cells. This recognition further stimulates their differentiation into plasma cells and the production of antibodies [27]. Additionally, the extracellular antigens can also be taken up by antigen-presenting cells (APCs) and digested by lysosomes into small fragments of antigens, which are subsequently presented to helper T cells via major histocompatibility complex II (MHC II) [28]. These helper T cells provide stimulatory signals, including recombinant cluster of differentiation 40 ligand (CD40L) and cytokines, to trigger B cell activation. They promote their differentiation of B cells into antibody-producing plasma cells and memory B cells while also activating phagocytes to clear pathogens through the production of inflammatory factors [29]. These antibodies target infected cells, facilitating the body’s immune responses and clearance of pathogens.

### 1.4. Feasibility of circRNA as a Vaccine Vector

Vaccines have played a critical role in the human response to various diseases. Nucleic acid vaccines represent the latest generation of vaccines, offering advantages such as stability, durability, and biodegradability. Consequently, they have become a major focus in vaccine research. Among these, mRNA vaccines emerged as a new class following the development of DNA vaccines, contributing significantly during the SARS-CoV-2 pandemic [30]. The circRNA was previously regarded as a non-coding RNA with no translation capability. However, in 2017, its ability to encode and translate proteins was identified in *Drosophila* [31], providing a theoretical basis for the research and development of circRNA vaccines. Extensive studies have demonstrated that circRNA vaccines exhibit remarkable stability, elicit robust immune responses, and induce the production of substantial amounts of neutralizing antibodies [32,33,34]. These characteristics position circRNA vaccines as some of the most promising candidates in contemporary vaccine research. Furthermore, the application of circRNA technology in SARS-CoV-2 vaccines highlights its potential for broader utilization in the prevention and treatment of various other diseases [35,36].

In this review, we summarize the synthesis, delivery, advantages, and applications of circRNA vaccines. The aim is to fully understand the application and prospects of circular RNA vaccines in disease treatment, identify the shortcomings in their synthesis and delivery, and propose potential development trends for future research directions. This work serves as a valuable reference for researchers in the fields of circRNAs and vaccines.

## 2. Synthesis of circRNAs In Vitro

CircRNAs are covalently closed, circular molecules formed by pre-mRNAs during variable splicing, where the downstream 5′ segment splice site (splice donor) is connected to the upstream 3′ segment splice site (splice acceptor), thus forming a circular RNA molecule with a 3′-5′ phosphodiester bond at the back splice junction (BSJ) [37]. The synthesis of circRNAs with specific functions in vitro holds significant importance, especially considering the growing potential of circRNAs in gene therapy applications. This synthesis process can be divided into two main steps: the preparation of linear RNA precursors and circularization.

### 2.1. Synthesis of Linear RNA Precursors

#### 2.1.1. Chemical Strategies

Linear RNAs are primarily prepared using natural nucleoside triphosphate derivatives and phosphoramidites through synthetic machinery. RNAs synthesized using this method exhibit high purity and are commercially available. However, the length of the synthesized strands is typically limited to 70–80 nucleotides [38]. While it is feasible to connect multiple smaller RNA oligonucleotides in tandem using ligases to obtain medium-sized RNAs, the synthesis of large RNA molecules through chemical synthesis remains a significant challenge. Therefore, the development of a new synthetic strategy is necessary to overcome this limitation.

#### 2.1.2. Enzymatic Strategies

**Figure 3:**
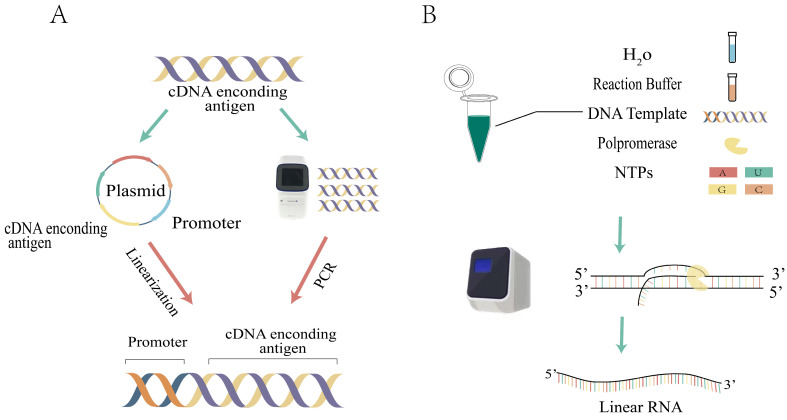
The process for the in vitro synthesis of linear RNA precursors involves several steps. (**A**) The production of DNA templates can be categorized into two approaches. The first is the construction of a plasmid vector containing the coding circRNA for the antigen, followed by amplification and linearization. Amplification is achieved using DNA polymerase chain reaction (PCR). (**B**) IVT reaction. The construction of the system using DNA as the template and NTPs as substrates and the properties of RNA polymerase. IVT, in vitro transcription.

The enzyme synthesis strategy involves the preparation of linear RNA precursors through an in vitro transcription reaction [39]. First, a DNA template must be prepared, which can be performed in two ways. The first method involves inserting the target sequence into a plasmid, linearizing the plasmid after amplification, and then obtaining a large quantity of the target sequence through enzyme digestion. The second method is to amplify the DNA template by PCR (Figure 3A). Following this, these DNA templates are transcribed using nucleoside triphosphates (NTPs) under the action of RNA polymerase and a promoter, ultimately producing linear RNA precursors (Figure 3B). The purity and quantity of the DNA templates play a crucial role in determining the RNA yield. This strategy enables the production of transcripts larger than 1 kilobase (kb) [39], addressing the limitation of the chemical synthesis method, which cannot be used to synthesize long RNA strands. However, it is important to note that the resulting RNA transcripts may not necessarily be identical to the natural transcripts in the organism, and this may also impact the biological activity of the product. Furthermore, the transcripts may be incomplete due to the RNA polymerase occasionally adding or missing a few bases at the ends during the in vitro transcription (IVT) reaction.

### 2.2. Circularization of RNA

The circularization of RNA represents a crucial step in circRNA synthesis. Currently, there are various strategies available for circularization, including chemical, enzymatic, and nuclease-based methods. However, both the chemical reagents and enzymes employed in these approaches often exhibit relatively low circularization efficiency. One commonly used strategy for circularization is the application of ribozymes, which leverages the fact that introns can self-splice to form a lasso-like structure. This ribozyme-based approach has gained popularity due to its effectiveness in achieving successful circularization.

#### 2.2.1. Chemical Strategies

**Figure 4:**
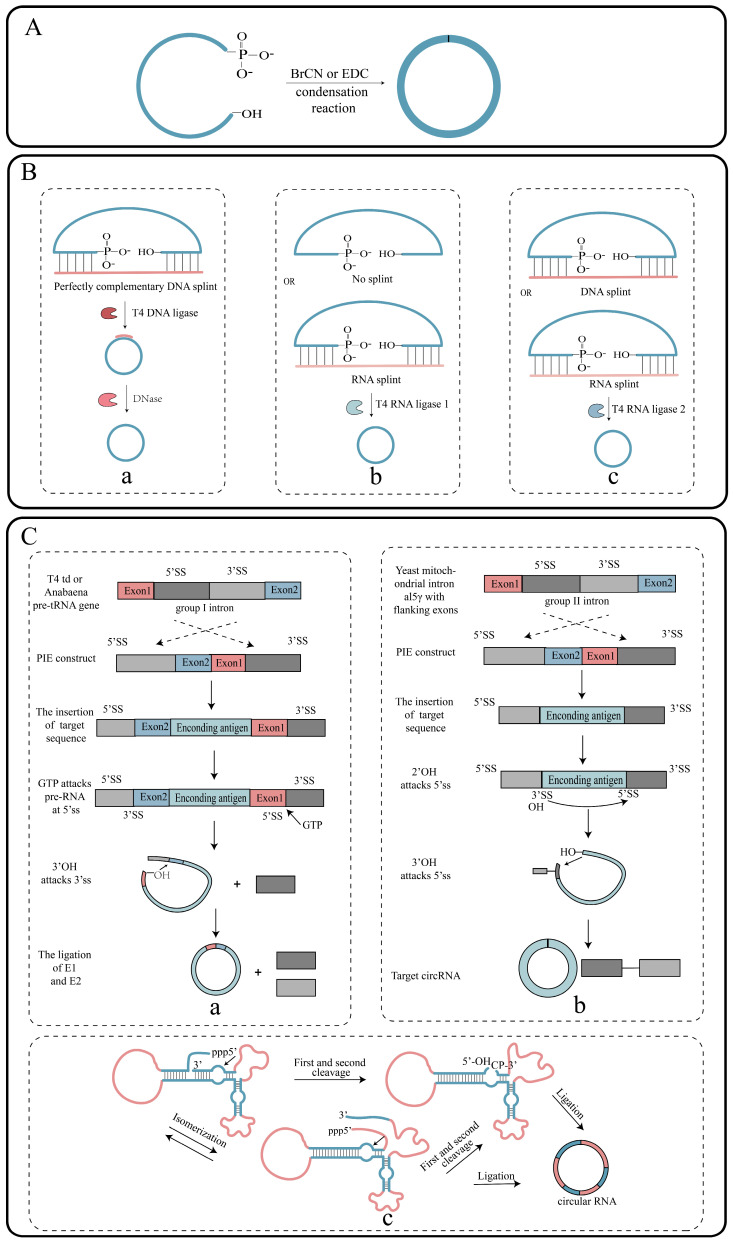
The in vitro circularization process of linear RNA precursors. (**A**) Chemical strategies for RNA circularization. (**B**) RNA circularization using T4 ligase; (**a**) The process of RNA circularization by T4 DNA ligase; (**b**) The process of RNA circularization by T4 RNA ligase I; (**c**) The process of RNA circularization by T4 RNA ligase II. (**C**) RNA circularization using ribozymes; (**a**) The mechanism of circularization of linear RNA precursors through self-splicing of group I introns; (**b**) The mechanism of circularization of linear RNA precursors through self-splicing of group II introns; (**c**) The process of circularization of linear RNA precursors using hairpin ribozymes.

The ligation of linear RNA can be achieved through the condensation of phosphate and hydroxyl groups at both ends of the RNA using chemical reagents such as cyanogen bromide (BrCN) or 1-ethyl-3-(3-dimethylpropyl) carbodiimide (EDC) (Figure 4A) [40]. However, the chemical method suffers from inefficiency, as well as the formation of a 2′-5′-phosphate bond instead of the desired 3′-5′ phosphodiester bond, making it unable to fully replicate the splice site found in vivo. Therefore, there are safety concerns associated with the use of chemical reagents, and their potential impact on biosafety remains unclear.

#### 2.2.2. Enzymatic Strategies

Ligases play a crucial role in linking the phosphate groups and hydroxyl groups at the ends of linear RNAs through the formation of phosphodiester bonds. Some enzymes commonly employed for linear RNA circularization are T4 DNA ligase 1 (T4-Dnl-1), T4 RNA ligase 1 (T4-Rnl-1), and T4 RNA ligase 2 (T4-Rnl-2), which are derived from the T4 phage. T4-Dnl-1 utilizes ATP as a cofactor and requires the design of a DNA splint that is complementary to the RNA nick ends. This DNA splint forms a locally hybridized double-stranded structure with the RNA molecule, allowing T4-Dnl-1 to recognize and bind to it, facilitating the circularization process by connecting the two ends of the RNA molecule [41]. The DNA splint can be subsequently digested using DNase, and a polyacrylamide gel can be used to isolate the circular RNA product from the linear precursor RNA (Figure 4B). Similarly, T4-Rnl-1 is also ATP-dependent and can only utilize single-stranded nucleic acid molecules as substrates. It requires the close proximity of the 5′ phosphate and 3′ hydroxyl groups for successful circularization. This often necessitates the assistance of RNA secondary structures or the design of RNA splints to bring the two ends closer together, facilitating circularization. T4-Rnl-1 exhibits a preference for different ends, with a preference order of A, G, C, U for the 3′ end and C, U, A, G for the 5′ end (Figure 4B) [41]. Likewise, T4-Rnl-2 is ATP-dependent and exhibits high efficiency for double-stranded RNA. However, its efficiency is influenced by the secondary structure of the RNA. The intramolecular ligation efficiency is typically enhanced through the design of DNA or RNA splints. Notably, T4-Rnl-2 was utilized successfully to circularize single-stranded RNAs consisting of 104 nucleotides (Figure 4B) [42].

#### 2.2.3. Ribozyme Strategy

Ribozymes, a class of RNAs with enzymatic catalytic properties, are used in RNA circularization through techniques such as the permuted introns and exons (PIE) system and the use of hairpin nucleases. The PIE system is the most commonly used method for in vitro circular RNA synthesis. It is based on the self-splicing capability of group I and group II introns, which enables the connection and circularization of intermediate intron sequences. In the presence of GTP, the PIE system utilizing the T4 td gene or the tRNALeu precursor gene from *Anabaena* enables the self-circularization of sequences other than introns [43]. The group I intron splicing system relies on magnesium ions and GTPs as cofactors. Through a two-step ester exchange pathway, the intron sequences are excised from the precursor RNA, resulting in the formation of a ring-structured RNA molecule (Figure 4C) [44]. An end-to-end self-targeting and splicing (STS) reaction method was developed to synthesize circRNAs, utilizing the P1 helical structure of the Tetrahymena group I intron [45]. Furthermore, in 2018, Wesselhoeft et al. engineered the *Anabaena* group I self-splicing intron to efficiently circularize a wide range of RNAs of up to 5 kb in length in vitro, achieving nearly 100% circularization efficiency. This approach also enabled stable protein expression in eukaryotic cells [46]. Group II intron splicing is significant for plant, fungal, and yeast metabolism and has been associated with various genetic variations in bacteria [47]. In group II intron splicing, the introns self-splice from exons and ligate the resulting fragments (Figure 4C). Mikheeva utilized group II intron sequences from the yeast mitochondrial genome to prepare circular RNA in 1997 [48]. Group II introns only require the activation of magnesium ions for circularizing RNA molecules, and they exhibit higher precision in connecting RNA molecules. Moreover, no exogenous sequences are introduced during the RNA circularization process. However, the efficiency of preparing circular RNA using group II introns is low, making industrial-scale production challenging.

Hairpin ribozymes (HPRs) are naturally occurring small RNA fragments that possess the ability to catalyze RNA splicing and regulate cleavage and ligation processes [49]. They are derived from various sources, including virus-like particles (VLPs) and hepatitis C viruses (HCVs). The production of HPR involves a rolled-loop transcription reaction using single-stranded DNA as a template. This reaction generates a long, repetitive, linear RNA precursor that undergoes self-scission to yield small circular RNAs [50]. Studies have demonstrated that the presence of cofactors that stabilize secondary structures can enhance circRNA synthesis [51]. The hairpin nuclease strategy is particularly effective in generating small circRNAs with high efficiency (Figure 4C).

The group I intron splicing nuclease method is widely applicable for circRNA production. However, it is constrained by the secondary structure of the linear RNA precursor. On the other hand, group II introns offer the more precise ligation of linear RNA precursors. Nevertheless, the in vitro mechanism of group II introns is not yet fully understood. HPRs provide an efficient means of generating small circRNAs. However, one drawback is that the introduction of exogenous HPR sequences can lead to the instability of the resulting circRNA products.

## 3. Delivery System for circRNA Vaccines

The effective delivery of vaccines is a crucial step in enabling them to function within the body. Exogenous RNA molecules encounter two major challenges upon entering cells. First, the ubiquitous RNases present in cells can degrade RNA, impacting the stability of RNA-based drugs. Second, negatively charged RNA has difficulty crossing cell membranes directly or entering cells through endocytosis. Therefore, when designing drug delivery vehicles, it is essential to consider these factors and their implications for clinical drug delivery.

### 3.1. Lipid Nanoparticle-Based Delivery Systems

Liposomes are a typical vaccine delivery system with components similar to cell membranes, offering high biocompatibility and biodegradability. Lipid nanoparticles (LNPs) represent a new generation of liposomes, primarily composed of phospholipids, cationic lipids, cholesterol, and polyethylene glycol lipids [52]. LNPs effectively address the challenges of low nucleic acid drug stability and poor permeability, making them one of the most common systems for nucleic acid drug delivery. Researchers utilized novel LNPs to encapsulate nucleic acid drugs, significantly downregulating the cyclin-dependent kinase 4 (CDK4) gene in tumor cells and thereby regulating tumor cell proliferation [53]. Subsequent research has successfully employed LNPs to deliver mRNA vaccines targeting the novel coronavirus [54]. Currently, liposome-based LNPs play a crucial role in circRNA vaccine delivery. Qu et al. were the first to design and synthesize a circRNA vaccine targeting SARS-CoV-2, encapsulating circRNA-RBD in LNPs and delivering it to rhesus macaques (Table 1), resulting in the induction of neutralizing antibodies [34]. Newly developed circRNA vaccines for monkeypox virus (MPXV) [55] and liver cancer [56] have also utilized LNP encapsulation, showing promising therapeutic effects. Moreover, targeted drugs are essential for precision treatment; LNPs can be modified on their surface to enhance targeting capability. For example, a lymph node-targeting circRNA-mLNP vaccine platform was developed by modifying the LNP surface with mannose (Table 1). This platform was applied to rabies virus and SARS-CoV-2 circRNA vaccines, both of which elicited strong immune responses [57]. This is largely attributed to the mannose modification, which improves dendritic cells (DCs) uptake of LNPs and promotes antigen migration toward lymph nodes while also allowing LNPs to withstand the mechanical stresses of lyophilization. The safety of LNP delivery systems has been validated in large-scale populations, and their modifiability provides the potential to target specific cells, making LNPs highly promising for gene therapy.

### 3.2. Other Delivery Systems

In addition to lipid-based delivery systems, naked RNA, extracellular vesicles (EVs), and virus-like particles (VLPs) are also utilized for circRNA drug delivery. Naked RNA delivery has been employed in various in vivo studies. For example, luciferase-encoding circRNA was delivered to mice with different tumor types via intratumoral injection, detecting fluorescent signals in mouse models of melanoma, non-small cell lung cancer, and colon adenocarcinoma [58]. However, most cells, aside from APCs like DCs, struggle to uptake naked RNA. Exosomes, nanoscale vesicles secreted by cells, can fuse with the plasma membrane of target cells, allowing them to directly enter the cells. They carry active molecules such as proteins, nucleic acids, and lipids, which have low immunogenicity and exhibit greater stability in body fluids compared to LNPs, offering unique advantages in drug delivery. The RVG (rabies virus glycoprotein)-circDYM-EVs system was developed, utilizing exosomes as carriers to achieve nicotinic acetylcholine receptor-targeted overexpression of circDYM (Table 1), effectively alleviating depressive behaviors induced by chronic unpredictable stress [60]. However, exosomes lack standardized extraction methods, and those derived from different cell types and generations can exhibit variability. VLPs are nanoscale particles that can be used to deliver drugs formed by the assembly of viral structural proteins, which are more biocompatible. Jaffrey and Unti developed a system called Tornado, which enables efficient expression of circRNAs in cells by utilizing VLPs for delivery, ultimately facilitating sustained translation of circRNAs [59].

Currently, LNPs are among the most widely used delivery vectors and offer significant advantages in delivering genetic drugs, such as circular RNA vaccines. However, the existing delivery systems face challenges, including limited drug loading capacity and poor stability of commonly used LNPs. Therefore, further research is needed to optimize vectors, including LNPs, to enhance the stability and targeting of delivery systems. APCs are sparse in peripheral tissues, so improving the targeting of vaccine delivery systems can help reduce the required antigen dose. Additionally, the application of EVs in delivery is still in its early stages, and the production of VLPs remains challenging, necessitating further optimization. CircRNA drugs are increasingly becoming a focal point in the biomedical field, with the delivery system playing a crucial role in their clinical application. Moving forward, research on the delivery systems for circular RNA vaccines must continue to improve in order to achieve safer, more stable, and efficient delivery.

## 4. Advantages of circRNA Vaccines

### 4.1. Enhanced Stability of circRNA Vaccines

The stability of vaccines directly impacts their efficacy. The mRNA vaccines offer advantages such as short development cycles, simple manufacturing processes, and the absence of viral components, making them prominent candidates due to their remarkable performance during the COVID-19 pandemic [65,66]. However, once inside the cytosol, mRNA can be degraded by RNase, particularly RNase R, within hours. In contrast, circRNA, with its covalently closed-loop structure, exhibits high stability and resistance to degradation by exonucleases (Table 2) [67]. 

The stability of circRNAs ensures continuous antigen expression in the body, thereby enhancing the immune response. Compared to linear mRNA vaccines, circular RNA can provide prolonged antigen expression over time [46]. For instance, the circRNA vaccine for liver cancer developed by Wang et al. has demonstrated more persistent antigen expression compared to mRNA vaccines [56]. CircRNA can lead to a higher accumulation of antigen proteins in the body, potentially aiding in the formation of long-lasting immune memory. Additionally, it is expected to achieve effective therapeutic outcomes while reducing the number of vaccinations needed, which helps lower production costs. Moreover, the translation of circRNAs is primarily driven by IRES or IRES-like elements [68]. During disease treatment, when the body is under stress, such as viral infection or cell apoptosis, the translation initiation of cap-dependent linear mRNA is inhibited [69], reducing the efficacy of mRNA-based drugs. In contrast, the translation of non-cap-dependent circRNAs continues normally, ensuring the efficacy of these therapeutic agents.

### 4.2. Induction of Robust Cellular Immunity

Cellular immunity, mediated by T cells, is essential in antiviral infection, antitumor immunity, immunomodulation, and transplant rejection, serving as a key immune defense mechanism in the body and also stimulating humoral immunity. Among the most promising vaccine candidates, circRNA vaccines, such as the COVID-19 vaccine, Monkeypox vaccine, and hepatocellular carcinoma vaccine [34,55,56], have demonstrated the ability to elicit a robust cellular immune response (Table 2). In a study, splenic cells from mice injected with a circRNA vaccine encoding the receptor binding domain (RBD) antigen of SARS-CoV-2 spike protein were collected and stimulated with SARS-CoV-2 RBD-Delta pooled peptides, resulting in increased levels of interferon-γ (IFN-γ), tumor necrosis factor-α (TNF-α), and interleukin-2 (IL-2) [34]. This suggests that the circRNA-RBD-Delta vaccine can effectively induce a potent cellular immune response. Additionally, a circRNA vaccine for monkeypox developed was also reported to induce a similar response [55]. Four circRNAs were designed encoding monkeypox virus proteins and injected them into mice. Fourteen days after the secondary immunization, splenic cells from vaccinated mice exhibited production of cytokines such as IFN, TNF, and IL in response to corresponding antigen peptides, compared to placebo-injected mice. Notably, the circMix4 combination vaccine and the circA35R vaccine triggered the most significant CD8^+^ T cell response. The researchers designed a circRNA encoding chick ovalbumin (circOVA) and used charge-altering releasable transporters (CART), CART-circOVA, and circRNA combined with chick Ovalbumin protein (OVAp) to immunize mice on days 0 and 7. The results showed that CART-circOVA induced an effective CD8 T cell response in the lungs and spleen by day 7. Furthermore, by day 42, the researchers observed that CART-circOVA not only induced a significant CD8 T cell response but also resulted in a 6 to 8 times higher proportion of CD8 T cells compared to the circRNA+OVAp group [70], suggesting that circRNA-encoded antigens can induce a more robust cellular immune response in vivo compared to protein antigens. This highlights the considerable advantage of circRNA as a vaccine carrier. The strong cellular immune response induced by circRNA vaccines indicates significant potential for combating pathogenic microbial infections and tumor treatments.

### 4.3. Induction of Robust Humoral Immunity

Neutralizing antibodies, a vital subset of immunoglobulins produced by B cells in response to pathogen invasion, such as by viruses and bacteria, serve as a critical measure of vaccine efficacy and community immunity. These antibodies have the unique ability to swiftly recognize and bind to the surface antigens of pathogens, effectively preventing their entry into cells and subsequent infection. In addition to neutralizing antibodies, binding antibodies are also produced, but only neutralizing antibodies possess the capacity to provide immediate defense against pathogens [71,72]. Multiple studies have demonstrated that circRNA vaccines can effectively induce a significant proportion of neutralizing antibodies (Table 2) [32,35,62]. Researchers successfully developed a circRNA vaccine targeting the novel coronavirus and found that the Omicron-specific circRNA vaccine induced high levels of neutralizing antibodies specifically against the Omicron variant. Similarly, the Delta-specific circRNA vaccine induced high levels of neutralizing antibodies against both the Delta and Omicron variants [34]. Moreover, the circRNA vaccine encoding the novel coronavirus VFLIP-X and the circRNA–mLNP–G vaccine have both demonstrated the ability to induce high titers of neutralizing antibodies against viral infections [57,62]. The circRNA vaccines demonstrate significant advantages in their ability to induce neutralizing antibodies. Studies have shown that, compared to the same dose of mRNA vaccines, circRNA vaccines can elicit higher levels of neutralizing antibodies [34,57], making them a preferred option for the prevention and treatment of various challenging diseases. Their potential applications extend to viral infections [73], tumors [74], and pathogenic bacterial infections [75].

## 5. Application of circRNA Vaccines in Prevention of Infectious Diseases

**Figure 5:**
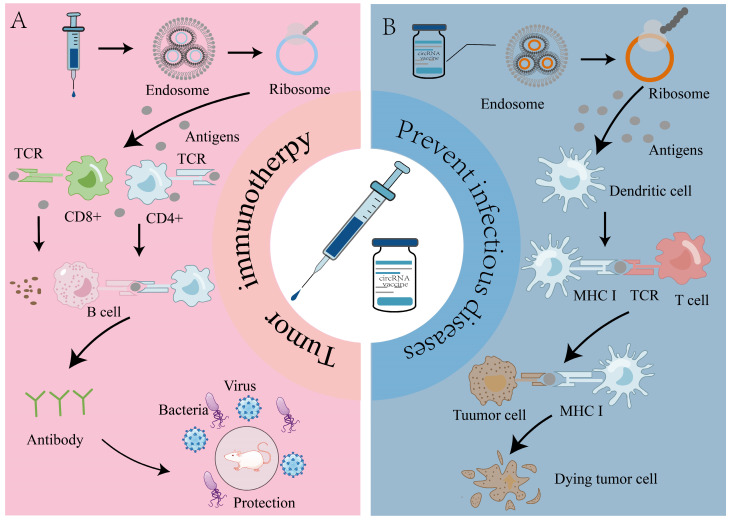
The application of circular RNAs in infectious disease prevention and tumor therapy. (**A**) Application of circRNA vaccines in the prevention of infectious diseases. The antigen encoded by a circRNA is presented to CD8^+^ T cells, leading to the production of cytokines or chemokines. Alternatively, it can be presented to CD4^+^ T cells, which in turn are recognized by B cells, leading to the production of antibodies that are immune to pathogenic microorganisms. (**B**) Application of circRNA vaccines in tumor therapy. Antigens encoded by circRNAs are recognized by DCs and presented to T cells. Subsequently, T cells present these antigens to B cells, leading to the production of antibodies that target tumor cells, ultimately resulting in the death of tumor cells. DCs, dendritic cells.

The prevention of epidemics is most effectively achieved through vaccination, which not only reduces the severity of diseases but also decreases the mortality rates and enhances social immunity, thereby controlling the spread of diseases, particularly in the context of mass vaccination campaigns [76,77]. By introducing exogenous antigens to stimulate the immune system, vaccination plays a crucial role in disease prevention by triggering the production of antibodies and memory cells (Figure 5A). mRNA vaccines have emerged as alternatives to traditional vaccines due to their simplified manufacturing process, shorter development cycle, strong immunogenicity, and high safety and efficacy [78]. However, mRNA vaccines exhibit challenges such as low stability, strict storage requirements, and potential side effects. It has been reported that circular RNA vaccines are more stable and can elicit stronger immune responses compared to mRNA vaccines [34]. A circRNA vaccine targeting the novel coronavirus was tested in rhesus macaques. A circular RNA encoding the SARS-CoV-2 spike trimeric RBD antigen was designed, delivered using LNPs, and subsequently infected the macaques with the virus seven weeks after immunization. Lung tissue samples were collected three days later for virus detection. The results demonstrated significantly lower viral loads in the circRNA vaccine group compared to the placebo group. Furthermore, the circRNA vaccine exhibited higher and more persistent antigen production and elicited a greater proportion of neutralizing antibodies compared to the 1mJ modified mRNA vaccine [34]. Another research team successfully developed a circRNA vaccine with neutralizing activity against SARS-CoV-2 and its variants, utilizing the VFLIP-X spiking protein of SARS-CoV-2. This vaccine was tested in mice and exhibited strong cellular and humoral immune responses. VFLIP-X immunization achieved a balanced response between T helper 1 (TH1) cells and T helper 2 (TH2) cells, with detectable neutralizing antibodies persisting seven weeks after injection [62]. The application of circular RNA technology to the development of SARS-CoV-2 vaccines offers renewed hope for the prevention of other infectious diseases. Additionally, a circRNA vaccine against *Staphylococcus aureus* has been developed, which induces immune responses in T and B cells by encoding bacterial immunodominant epitopes [79]. This vaccine represents a novel strategy for the treatment of bacterial infections, aiming to replace antibiotic therapy, reduce the reliance on antibiotics, and mitigate the issue of drug resistance. Recently, a circRNA vaccine targeting the monkeypox virus (MPXV) triggered a strong humoral and cellular immune response in mice and effectively protected immunized mice from being attacked by the MPXV [55]. This study demonstrates the potential of circular RNA vaccines in preventing monkeypox virus and provides new directions for future vaccine development.

These circRNA vaccines address the limitations of mRNA vaccines, such as stability issues and stringent storage requirements, positioning them as highly promising alternatives to mRNA vaccines. Clinical trials for mRNA vaccines targeting respiratory syncytial virus (RSV), influenza virus, rabies virus, human immunodeficiency virus, cytomegalovirus, and SARS-CoV-2 are already underway [80,81,82,83,84]. In these areas, circRNA vaccines also hold tremendous potential.

## 6. Role of circRNA Vaccines in Tumor Therapy

Tumors have long presented significant challenges to human health. While surgical interventions, chemotherapy, and radiotherapy are commonly employed, they can cause substantial harm to the body and carry the risk of tumor recurrence. In recent years, immunotherapies have emerged as a promising approach to cancer treatment, including tumor vaccines [85], chimeric antigen receptor T cell immunotherapy (CAR-T) [86], and immune checkpoint therapies [87]. Tumor vaccines aim to activate the patient’s cellular and humoral immune responses by targeting tumor-associated antigens (TAAs) and tumor-specific antigens (TSAs), resulting in the elimination of tumor cells (Figure 5B) [88]. Researchers have achieved significant advancements in this field by developing a circRNA vaccine encoding tumor antigens. This vaccine, encapsulated in LNPs (composed of ionizable lipids that induce proinflammatory factors in the LNPs), stably expresses tumor antigens while activating cytotoxic T cells. It successfully induced potent immune responses in various mouse tumor models and effectively inhibited melanoma progression [33]. Chen et al. showed that circRNA vaccines encoding H19-IRP (a TAA) induced a robust cytotoxic T cell response, effectively inhibiting the growth of glioblastoma (GBM) [89]. Furthermore, circFAM53B has been identified, a circRNA encoding TSAs that is uniquely expressed in breast cancer but absent in normal breast tissue and cell lines. This circRNA promotes DC function and activates CD8^+^ T cells, leading to the inhibition of tumor growth and migration [90]. This discovery provides a foundation for the exploration of the non-canonical translation of TSAs from circRNA for cancer immunotherapy. A recent study developed a circRNA vaccine targeting hepatocellular carcinoma (HCC)-specific tumor neoantigens. This research shows that, compared to linear RNA vaccines, this circRNA vaccine offers the advantage of stable and persistent expression of antigen proteins, significantly promoting the activation of DC cells and T cells in vitro. Additionally, this circRNA vaccine exhibits anti-tumor efficacy that surpasses that of linear RNA vaccines in both subcutaneous and orthotopic murine HCC models. Furthermore, it shows superior tumor prevention effects compared to linear RNA vaccines [56]. Additionally, a recent study demonstrated that the combination of circRNA DC vaccine and gemcitabine (Gem) effectively inhibited the growth of pancreatic tumors in mice, achieving an inhibition rate of 89% [36]. mRNA vaccines have shown great potential in cancer immunotherapy due to their ability to generate effective immune responses, their ease of production, and their relatively minor side effects [91]. Considering the capacity of circRNAs to encode antigens and address the limitations of mRNA vaccines, such as stability issues and short protein expression durations, circRNA vaccines offer broader potential in tumor treatment. Tumor vaccines targeting a limited number of antigens have limitations in treating heterogeneous tumors. Significant advancements in cancer treatment have been achieved by combining tumor vaccines with CAR-T, T cell receptor gene-engineered T cells (TCR-T), and programmed cell death protein 1 (PD-1) therapies. These combination approaches have yielded remarkable progress in cancer therapy [92,93].

### 6.1. Application of circRNA Vaccines in CAR-T and TCR-T Therapy

CAR-T and TCR-T cell therapies represent innovative approaches in cancer immunotherapy. CAR-T therapy involves isolating T lymphocytes from patients’ blood and expanding and genetically modifying them in vitro to express receptors that target tumor cell surface antigens, thereby enabling T cells to recognize and eliminate tumor cells. While traditional CAR-T therapy is complex, time-consuming, and costly, the concept of novel CAR-T therapy has emerged as a potential treatment platform. Novel CAR-T therapy involves delivering antigen-encoding RNA drugs into the body to reprogram T cells and embed tumor antigen receptors on their surface membranes, enabling them to express tumor antigen receptors and achieve cancer treatment. Hu et al. established a mass-produced, scarless circRNA preparation platform based on the PIE system, constructed T cells based on circRNA expression of anti-CD19 CAR (cmCAR-T), and verified its therapeutic effect on tumors in both in vitro and in vivo models. Additionally, compared with linear mRNA, cmCAR-T cell therapy has greater advantages in tumor treatment [94]. CAR-T therapy has demonstrated remarkable efficacy in hematological malignancies, including acute lymphoblastic leukemia and non-Hodgkin’s lymphoma [95,96]. However, its application in solid tumors is limited by the antigens’ heterogeneity and loss. Recent research suggests that the combination of vaccines with CAR-T therapy can effectively address solid tumors with antigen heterogeneity. Vaccine-enhanced CAR-T cells produce large amounts of IFN-γ, thereby activating a large number of T cells, which work together to circumvent the escape of antigen-negative tumor cells, thereby achieving tumor cell eradication and preventing tumor regeneration [97]. A strategy combining RNA vaccines with CAR-T therapy targeting the embryonic tumor antigen CLD6 has yielded promising results in patients [98]. Researchers prepared circRNA encoding chimeric antigen receptor (CAR) (circRNA-CAR) in vitro and successfully expressed it in T cells and macrophages, demonstrating effective killing of tumor cells in vitro. After injecting it into mice, they found that circRNA-CAR improved the survival rate of mice with tumors. Additionally, the study revealed that the combination of the anti-human epidermal growth factor 2 (HER2) circRNA-CAR (referred to as circRNA-Anti-HER2-CAR) and a circRNA vaccine encoding HER2 antigen elicited a strong anti-tumor immune response [99].

TCR-T therapy, another personalized treatment approach, involves extracting tumor antigen-specific T cells from patients, genetically modifying and expanding them in vitro, and reintroducing them into patients to eliminate cancer cells [100]. TCR-T therapy has shown potential in treating various cancers, including melanoma, non-small-cell lung cancer, and cervical cancer [101]. However, challenges such as the short-term persistence of T cells in vivo and the risk of tumor antigen escape remain. A recent study discovered that DCs transfected with circRNA encoding the cytomegalovirus pp65 antigen effectively selected antigen-specific T cells in vitro, demonstrating higher efficiency compared to those transfected with mRNA [102]. Cytomegalovirus (CMV) infection is a significant cause of morbidity and mortality in patients undergoing hematopoietic stem cell transplantation [103], making its prevention crucial in curing hematological tumors. CircRNA vaccines, as highly promising RNA vaccines, have shown success in TCR-T cell therapy, offering new, safer, and more effective treatment approaches and strategies for cancer therapy.

### 6.2. CircRNA Vaccine Used in Combination with PD-1 for Tumor Treatment

The PD-1 monoclonal antibody is a drug that inhibits the binding of PD-1 on the surfaces of T cells to PDL-1 on the surfaces of tumor cells. This mechanism prevents tumor cell escape and reactivates the immune response of T cells against tumor cells [104]. PD-1 has been found to play a crucial role in various cancers, including breast, lung, colorectal, and gastric cancers [105,106,107,108]. However, PD-1 therapy is associated with drug resistance and low response rates in patients. The combination of PD-1 therapy with tumor vaccines has emerged as a promising strategy to address these challenges. Several clinical trials are investigating the combination of tumor vaccines with PD-1 therapy. In a mouse model, some researchers observed that injecting circRNA encoding a mixture of cytokines induced an anti-tumor immune response and enhanced PD-1-mediated tumor control [58]. mRNA cancer vaccines have also shown effectiveness in combination with PD-1 therapy [109]. CircRNA vaccines have emerged as a promising alternative to mRNA vaccines due to their high stability, prolonged protein expression, and ability to induce robust cellular and humoral immunity. The combination of circRNA vaccines and PD-1 therapy holds great potential in oncology and is expected to become a prominent trend in the future for the treatment of solid tumors.

## 7. Conclusions and Prospects

Circular RNAs (circRNAs) possess greater potential as vaccines compared to mRNAs due to their stability, their ability to encode proteins, and their capacity to induce a large percentage of neutralizing antibodies. CircRNA vaccines offer significant advantages in combating infectious diseases such as SARS-CoV-2 and targeting tumors. In fact, circRNAs have been declared to be the most promising alternatives to mRNAs [110]. However, several challenges still need to be addressed before the practical application of circRNAs in disease treatment.

The process of synthesizing circRNA molecules in vitro involves designing sequences encoding corresponding antigens, preparing DNA templates, and performing in vitro transcription and circularization. Currently, a major challenge is the low efficiency of the in vitro circularization of linear RNA. Chemical and enzymatic reagents exhibit very low efficiency in circularizing linear RNA, posing challenges for circRNA synthesis. This efficiency problem also affects the purification of circRNAs, which directly impacts vaccines’ efficacy. Uncircularized linear RNA and residual enzymes can impact the immune response, potentially affecting the recognition of pattern recognition receptors and antigens. Existing purification strategies, such as the RNase R digestion of linear RNA or high-performance liquid chromatography (HPLC) purification, are costly (Table 2) and unsuitable for mass production in factories. Moreover, the delivery system for circRNA vaccines plays a critical role. Currently, liposomes are primarily used for delivery, but further improvements are needed to enhance their targeting capabilities. Achieving the accurate delivery of circRNA vaccines to the target tissue is particularly important for tumor therapy. Additionally, the limited length of the antigens encoded by circRNAs restricts their function as protein carriers when encountering large antigen fragments.

To overcome these challenges, it is essential to develop new technologies and approaches and improve the efficiency and purity of circRNA circularization. Advancements in synthetic biology are crucial for the development of circRNA vaccines. Furthermore, it is necessary to enhance the targeting capabilities of circRNA vaccine delivery systems to achieve the precise release of circRNA vaccines.

## Figures and Tables

**Table 1:** Delivery systems for RNA vaccines.

System Type	System Name	Mechanisms	Advantages	References
Naked RNA delivery	Intratumoral injection	The naked RNA is taken up by DCs through micropinocytosis.	Avoid side effects Good targeting	[58]
VLPs (virus-like particles)	VLP	VLP possesses viral structural proteins but lacks a viral genome, and its mechanism of entering cells is consistent with that of natural viruses.	Targeted delivery Good safety	[59]
Extracellular vesicle	EV	Exosome fuses with the plasma membrane of the target cell or is endocytosed directly into the cell to release the payload.	High tissue penetration Good biocompatibility	[60,61]
LNPs	LNP	LNP is taken up via endocytosis. The pH of the mature endosome decreases, causing the LNP to become protonated, which leads to the fusion with the endosomal membrane and the release of circRNA.	Simple preparation High effectiveness Large load	[33,34,56,62]
	TG6A-LNP	TG6A (an ionizable glycerolipid) undergoes rapid degradation upon entering the cytoplasm, releasing the circRNA.	Excellent degradability High protein expression	[63]
	H1L1A1B3 LNP	H1L1AB3 is a tumor-targeting ionizable lipid that can directly release drugs to the tumor site.	High transfection efficiency	[64]
	mLNP	By modifying ionizable lipids with mannose, DCs can take up nanoparticles via mannose receptor-mediated endocytosis.	Stable targeting Stable physical properties	[57]

Abbreviations: DCs, dendritic cells; VLP, virus-like particle; LNP, lipid nanoparticle; EV, extracellular vesicle; circRNA, circular RNA; pH, potential of hydrogen; mLNP, mannose lipid nanoparticle.

**Table 2:** Advantages and disadvantages of circRNA vaccines.

	Advantages	Disadvantages
Stability	High stability and resistance to RNase hydrolysis	Unknown
Security	Contains no virus ingredients, no risk of infection	Unknown clinical safety issues
Immunogenicity	It can induce both humoral immunity and high proportion of neutralizing antibodies	Potential adverse effects caused by immunogenicity
Production	No complicated modifications required	High production cost and unstable cyclization efficiency

## Data Availability

Not applicable.
